# Associations Between Dietary Inflammatory Index and Sex Hormones Among 6- to 19-Year-Old Children and Adolescents in NHANES 2015–2016

**DOI:** 10.3389/fendo.2021.792114

**Published:** 2022-01-10

**Authors:** Yuxia Ma, Ruiqiang Li, Wenqiang Zhan, Xin Huang, Yutian Zhou, Yan Sun, Hao Tian, Huichen Zhu, Bowen Yin

**Affiliations:** ^1^Department of Nutrition and Food Hygiene, School of Public Health, Hebei Province Key Laboratory of Environment and Human Health, Hebei Medical University, Shijiazhuang, China; ^2^School of Public Health, Shanghai Jiao Tong University School of Medicine, Shanghai, China

**Keywords:** Dietary Inflammatory Index, sex hormones, children, adolescents, inflammation

## Abstract

**Objectives:**

This study aimed to assess the relationship between dietary inflammatory index (DII) and sex steroids in children (6-11 years old) and adolescents (12-19 years old) in the U.S. National Health and Nutrition Examination Survey, 2015–2016.

**Methods:**

Participants between the ages of 6-19 have 24-hour dietary intake data, serum sex hormones [total testosterone (TT), estradiol (E2)], and sex hormone-binding globulin (SHBG) available data (n = 1382). The free androgen index (FAI) is calculated as TT divided by SHBG and the ratio of TT to E2 (TT/E2). The constructed puberty state is defined as high levels of steroid hormones (TT≥50 ng/dL in men, E2≥20 pg/ml in women) or onset of menarche. Multiple linear regression analysis was stratified by gender-age and gender-pubertal status groups to evaluate the association between DII and sex hormone levels.

**Results:**

After adjusting for covariates, the association between consecutive DII and sex hormone indicators by gender and age group. In male adolescents, DII was always negatively associated with TT (P-trend = 0.09), FAI (P-trend = 0.03) and E2 (P-trend = 0.01), and monotonically positively associated with SHBG (P-trend = 0.02).In female adolescents, with the increase of DII, a significant positive correlation with SHBG was observed (β 0.017, 95%CI: 0.009,0.053) (Table 3). Among female adolescents, a significant negative association between DII and TT and a significant positive association between SHBG were observed in this group. Moreover, DII was positively associated with SHBG of prepubertal males and negatively associated with FAI of prepubertal females.

**Conclusions:**

DII was associated with decreased levels of certain sex steroid hormones (TT, FAI, and E2) and increased levels of SHBG in adolescents or pubertal individuals, with the associations presenting somewhat sex-dependent pattern. However, there is little evidence that there is a significant association in children or prepubertal children. Further research needs to be carried out to verify our results.

## Introduction

More and more studies have shown that diet may be related to inflammation markers (1). Dietary patterns allow assessment of the complex interactions of food nutrients and health and may be associated with inflammatory status (2). Eating patterns characterized by higher intakes of red and processed meats, peas, beans, and fried foods, and lower intakes of whole grains are associated with higher inflammatory markers (3). Meanwhile, a diet rich in fruits and vegetables has been recommended to combat oxidative stress and inflammation, and there have been studies showing that eating more fruits and vegetables will lead to a decrease in pro-inflammatory mediators and an enhancement of the immune cell spectrum (4, 5).

It is known that dietary ingredients affect the chronic low-grade inflammation state (6). The Dietary Inflammatory Index (DII^®^) is applied to assess an individual’s potential for dietary inflammation (7). It was created by assigning a score to each of 45 nutrients and food ingredients, which are reported to affect the levels of inflammation markers, such as CRP, IL-1b, IL-4, IL-6, IL -10, and TNF-a, using articles from more than 1,900 peer-reviewed journals (8). In cross-sectional and longitudinal studies, the index has been verified by the relationship between it with multifarious inflammatory markers, including CRP, IL-6, and TNF-α (9).

Previous studies have found that several common dietary indexes are associated with the concentration of sex hormones (10, 11). The diet quality measured by the Alternative Healthy Eating Index (AHEI) is negatively correlated with premenopausal estrogen concentration (12). Inflammatory diet intake may also have an impact on sex hormones, and more pro-inflammatory diets are associated with lower TT and E2 levels in male adolescents (13). Moreover, the high intake of certain oxidants and pro-inflammatory dietary components (such as polyunsaturated fatty acids) is related to the level of sex hormones (14). The dietary inflammatory index can measure an individual’s dietary inflammation potential (15). A higher DII score can predict Increased levels of inflammation markers (including interleukin 6 and c-reactive protein) (16–18).

There has been little research on the association between DII and sex steroids in children and adolescents, and the proportion of pro-inflammatory diets in children and adolescents is gradually increasing in contrast to adults, so they may be more susceptible to the dysgenic influences of dietary inflammatory potential on many vital developmental terminal points. Given the different effects of sex hormones on sexual maturity in children and adolescents, it is necessary to explore the relationship between DII and sex hormones at two different developmental stages (19). Hence, this study aims to evaluate the association between DII and serum sex steroids in 6-19-year-old children and adolescents based on data from the National Health and Nutrition Examination Survey (NHANES, 2015-2016).

## Methods

### Study Design and Population

**Figure 1:**
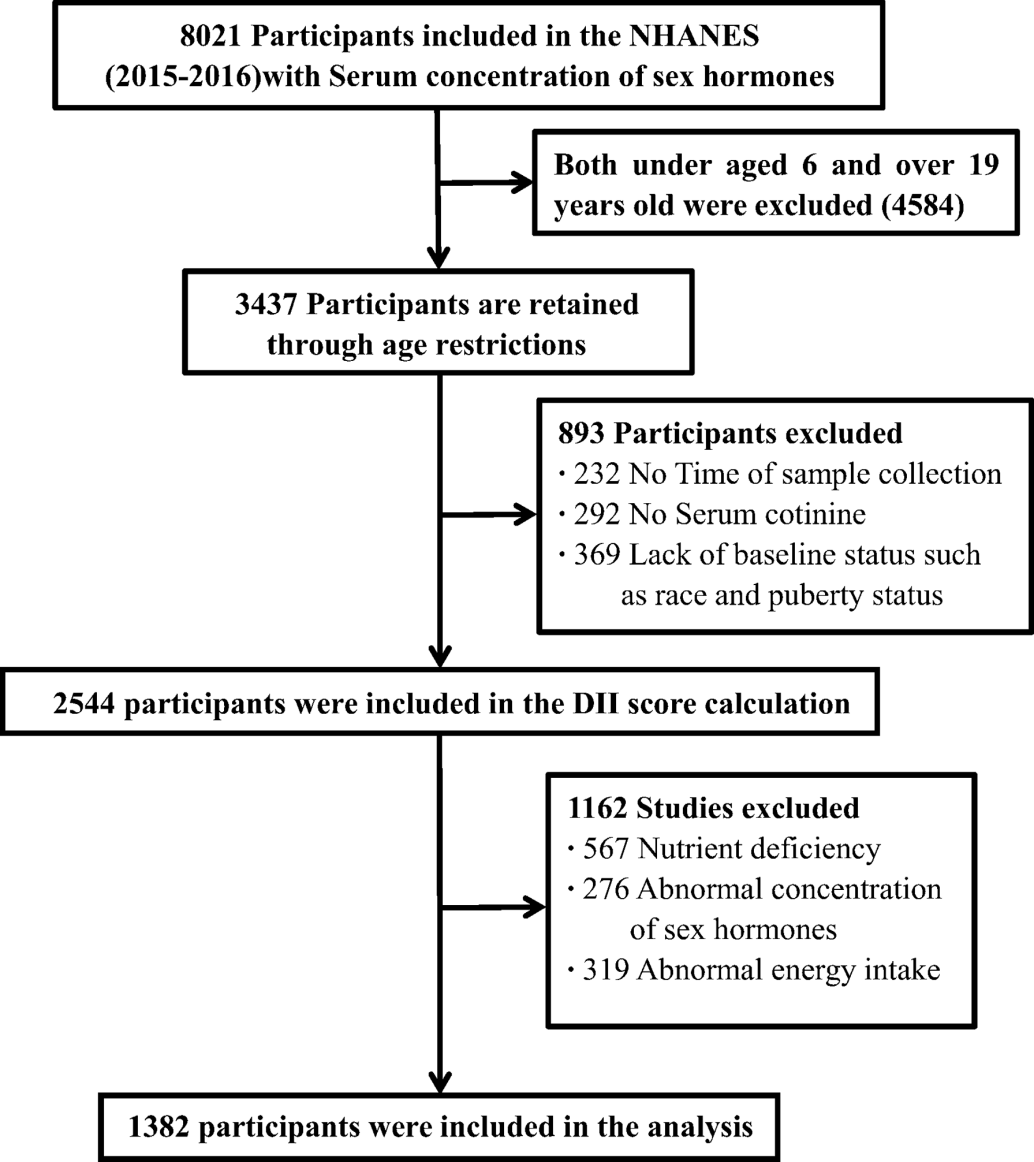
Flowchart of the sample selection in NHANES 2015-2016.

Our research is a cross-sectional study based on the National Health and Nutrition Examination Survey (NHANES) database. The National Health and Nutrition Examination Survey (NHANES) is a nationally representative survey conducted by the National Center for Health Statistics (NCHS) attaching to the Centers for Disease Control and Prevention (CDC). NHANES is an ongoing national cross-sectional survey of the United States that uses a complex multistage sampling strategy and is conducted every two years by the National Center for Health Statistics at the Centers for Diseases Control and Prevention. The cross-sectional survey included measuring the health and nutritional status of the American population, as well as the chemical substances and metabolites in the blood and urine. In this study, a total of 1382 participants aged 6-19 years were selected from the 2015-2016 NHANES, who had a 24-hour dietary recall of dietary intake, serum total testosterone (TT), and estradiol (E2) and the available data of sex hormone-binding globulin (SHBG) (**Figure 1**).

When analyzing data from complex surveys, sampling weights are usually used to generate representative and unbiased statistical data. However, if the variables used to calculate sampling weights (such as race, age, and income) are further adjusted in the regression analysis, it will reduce the accuracy of the estimation and even introduce excessive adjustment bias to a certain extent (20). Therefore, we show our results without sampling weights, similar to some previous studies using NHANES data (21, 22).

### Exposure and Outcome Definitions

There are some studies on the development and verification of DII in detail. Research from more than 1900 peer-reviewed publications forms the basis of DII. The “Inflammatory Effect Score” was created from these peer-reviewed publications for each DII food parameter, based on their impact on inflammatory cytokines. At the same time, standardize DII calculations into a world database with regional representation. This world database includes the dietary consumption of 11 people from all over the world (7). The world database provides standard averages and deviations of all DII food parameters. For each food parameter, create a z-score by subtracting the individual’s estimated intake from the standard average. It is then divided by the world standard deviation and converted to a distribution centered at 0 and bounded between -1 and +1. This value is then multiplied by the inflammatory effect score for each food parameter, and then all food parameters are added together to create an overall DII score. The more positive scores mean the more pro-inflammatory diet, the more negative values, the stronger the anti-inflammatory effect (7). In this study, 27 of the 45 food parameters can be obtained through NHANES data, including grams of alcohol, protein, fiber, total fat, carbohydrates, cholesterol, omega3, and omega6, saturated fat/MUFA/PUFA, magnesium, niacin, zinc, Fe, riboflavin, folic acid, β-carotene, caffeine, selenium, thiamine and vitamins A, B_6_, B_12_, C, D and E. DII scores range from negative tail to positive tail, more negative values indicate anti-inflammatory properties and corrected scores indicate pro-inflammatory properties. Energy adjusted DII (E-DII) food intake per 1,000 calories is used to explain the effect of total intake on energy intake (7, 23–25).

Serum samples are processed at −20°C and stored in vials until they are shipped to the National Environmental Health Center for testing. Serum TT and E2 levels are measured by using isotope dilution high-performance liquid chromatography-tandem mass spectrometry (ID-LC-MS/MS), and to quantify the concentration of SHBG in line with the response of SHBG with immune antibodies and chemiluminescence measurement of the reaction product through photomultiplier tube. The technical details of these methods are described elsewhere. The lower limit of detection (LLOD) of TT, E2, and SHBG are 0.75 ng/mL, 2.994 pg/m, and 0.800 nmol/l, respectively. We also computed the free androgen index (FAI), which is the value of TT (ng/dL) divided by SHBG (nmol/L) and the ratio of TT to E2 (TT/E2) to mediately evaluate the proximate amount of circulating free testosterone and aromatase activity, respectively (26, 27).

### Dietary Inflammatory Index

The calculation of the dietary inflammatory index links the personal dietary data obtained in each clinical study with the global average intake. The specific formula is Z score = (daily intake of this kind of dietary ingredient or nutrient-this kind of dietary ingredient or the global average per capita daily intake of nutrients)/The standard deviation of the global average per capita daily intake of this dietary ingredient or nutrient. Then convert the Z score to a percentile system (to reduce the influence of outlier effects), double the obtained percentile value, and subtract “1” to achieve a symmetrical distribution centered on “0”. Finally, multiply by the total inflammatory score of each dietary component, and combine the results to obtain the personal dietary inflammatory index score (7). DII scores range from negative tail to positive tail, more negative values indicate anti-inflammatory properties and corrected scores indicate pro-inflammatory properties (28).

DII calculation formula:


$$\begin{array}{l} Zscore=[(\text{daily mean intake}\;-\;\text{global daily mean intake})/\text{standard deviation}]\\ Zscore^{1}=Zscore\to (\text{converted to a percentile score})\times 2-1\\ DII\;=\sum Zscore^{1}\times \text{the inflammatory effect score of each dietary component} \end{array}$$

### Study Covariates

Because the level of sex hormones varies in different genders and developmental stages, we performed descriptive statistics and stratified analysis of men and women by age group (children: 6-11 years old, and adolescents: 12-19 years old). It is worth noting that dividing 6-19-year-old participants into subgroups of children and adolescents according to age alone may result in a mixture of prepubertal and adolescent individuals. This will result in abnormally high or low levels of sex hormones in each subgroup, which may distort the association between DII and sex hormones in the regression analysis. In addition, depending on the state of puberty, the effect of DII on sex hormones may also be different. To solve this problem, we further divided the participants into puberty and prepubertal groups based on serum sex hormone levels and menarche status. If the participant’s TT ≥ 50 ng/dL (male) or E2 ≥ 20 pg/mL (female) or menstruation has started (female), the participant is classified as having entered puberty (i.e. adolescent group). Others are classified as not reaching puberty (i.e. prepubertal group). Information about menarche for girls is retrieved from two questions: “Has menstruation started?” (in the health status questionnaire) and “Age at the first menstrual period?” (in the reproductive health questionnaire). Women who answered “yes” or had data available for their first menstrual age were recorded as “start of menarche”.

According to previous research, the multivariate model contains latent variables that confuse the association between DII and sex hormones, which include age (continuous), race/ethnicity (category), body mass index (BMI) category, poverty income ratio (PIR, continuous), and cotinine (category), and sampling time set (category). NHANES calculated the BMI categories of children and adolescents aged 2-19 years, defined as four levels: underweight (BMI <5%), normal weight (5 ≤ BMI <85%), overweight (85 ≤ BMI <95%) and obesity (BMI ≥ 95%). Directed acyclic graphs (DAGs) were used to describe the relationships between potential confounders (**Supplementary Figure 1**). Due to the low number of people in the underweight category (n = 23), we combined underweight and normal weight into one category in the regression analysis. PIR is calculated by dividing the family income (based on the poverty criteria specific to the size of the family) by the year and state and is used to embody the socioeconomic status of the participant in the entire family. Race/ethnicity is coded as non-Hispanic white, non-Hispanic black, Hispanic (Mexican American and other Hispanics), etc. The biological sample collection time is coded as morning, afternoon, and evening.

### Statistical Analysis

In this study, continuous variables are expressed as mean standard deviations, while categorical variables are expressed as proportions. Calculate the difference between different DII groups (quartiles) by using the chi-square test (categorical variables) or t-test (continuous variables).To examine the association between DII and sex hormones, multivariate linear regression explored sex hormones as continuous variables. Since the relationship between age stage and puberty status and sex hormones of different genders has been well established, age stage and puberty status are regarded as pre-designated potential influence modifiers. An additional stratified analysis was performed by age stage and adolescent status category. Missing values are entered by the median (if continuous) or mode (if categorical) of the existing cases of the variable. All statistical analyses were performed using the software package R (http://www.R-project.org, The R Foundation). A two-tailed p-value of <0.05 was considered statistically significant.

## Results

**Table 1** shows the demographic characteristics of 1,382 NHANES children and adolescents grouped by age and gender collected from 2015 to 2016. The average age of the participants was 12.2 years (± 3.9 years). The overall detection frequencies of TT, E2, and SHBG are 100%, 63.6%, and 100%, respectively. However, the detection frequency of E2 in children and prepubertal participants is <50%. Therefore, E2 and TT/E2 analyses were not carried out in these groups. **Table 2** lists the sex hormone concentrations of the participants and ranks them by DII quartile. The average DII is 0.92 ± 0.33, and the score ranges from -5.42 (most anti-inflammatory) to +4.39 (most pro-inflammatory), the intake of each nutrient in DII is shown in **Supplementary Table 1**. The total testosterone concentration ranges from the highest quartile (most pro-inflammatory) of DII at 23.45 to the lowest quartile (most anti-inflammatory) at 16.20. The distribution of other hormones is shown in **Table 2**.

**Table 1:** Characteristics of 6-19-year old children and adolescents with serum sex hormones and urinary Dietary inflammatory index(DII) in NHANES 2015-2016.^1^

Characteristics	All (n = 1382)	Female		Male	
		Children (n = 322)	Adolescents (n = 389)	Children (n = 320)	Adolescents (n = 351)
Age [yrs, mean (SD)]	15.5 (2.1)	8.5 (1.7)	15.3 (2.2)	8.6 (1.7)	15.3 (2.3)
Race/ethnicity [n (%)]					
Mexican American	335 (24.2)	86 (26.71)	90 (23.14)	74 (23.13)	85 (24.22)
Other Hispanic	179 (13.0)	45 (13.98)	54 (13.88)	50 (15.63)	30 (8.55)
Non-Hispanic White	375 (27.1)	83 (25.78)	105 (26.99)	83 (25.94)	104 (29.63)
Non-Hispanic Black	287 (20.8)	64 (19.88)	71 (18.25)	73 (22.81)	79 (22.51)
Other/multi-racial	206 (14.9)	44 (13.66)	69 (17.74)	40 (12.50)	53 (15.10)
BMI Category [n (%)]^2^					
Underweight/Normal weight	792 (57.3)	194 (60.24)	209 (53.73)	190 (59.37)	199 (57.35)
Overweight	266 (19.2)	59 (18.32)	94 (24.16)	51 (15.94)	62 (17.87)
Obese	320 (23.2)	69 (21.43)	86 (22.11)	79 (24.69)	86 (24.78)
Time of sample collection [n (%)]					
Morning	595 (43.1)	128 (39.75)	183 (47.04)	113 (35.31)	171 (48.72)
Afternoon	512 (37.0)	120 (37.27)	135 (34.70)	126 (39.38)	131 (37.32)
Evening	275 (19.9)	74 (22.98)	71 (18.25)	81 (25.31)	49 (13.96)
Poverty income ratio [median (IQR)]	1.51 (0.92-2.89)	1.53 (0.74-2.81)	1.55 (0.92-2.89)	1.65 (0.86-3.14)	1.58 (0.83-2.79)
Serum cotinine [ng/mL, median (IQR)]	0.024 (0.011-0.099)	0.032 (0.011-0.104)	0.023 (0.011-0.0935)	0.024 (0.011-0.096)	0.040 (0.011-0.54)
Age at menarche [yrs, median (IQR)]^3^	12.0 (11.0-13.0)	12.0 (11.0-13.0)	12.0 (11.0-13.0)	–	–
“Menstrual period started yet?”^4^					
Yes	363 (51.1)	20 (6.2)	343 (88.2)	–	–
No	235 (33.1)	215 (66.8)	20 (27.5)	–	–
Missing	113 (15.9)	87 (27.0)	26 (6.7)	–	–
Serum sex hormones indices [median (IQR)]^5^					
TT (ng/dL)	18.70 (4.42-80.98)	4.64 (2.85-10.58)	24.5 (17.7-32.75)	3.67 (2.09-6.26)	388.0 (241.0-532.0)
E2 (pg/mL)	12.80 (2.11-33.88)	2.11 (2.11-16.80)	52.4 (28.6-13.5)	2.11 (2.11-2.11)	19.3 (11.9-25.8)
SHBG (nmol/L)	56.99 (34.89-97.05)	78.48 (48.10-114.10)	48.38 (33.04-75.37)	93.68 (61.00-136.10)	34.09 (22.37-48.70)
FAI	0.34 (0.056-2.05)	0.069 (0.028-0.190)	0.495 (0.310-0.789)	0.041 (0.0017-0.103)	12.43 (7.54-17.02)
TT/E2	1.39 (0.59-11.15)	1.02 (0.61-1.62)	0.51 (0.23-0.80)	1.71 (0.97-2.82)	19.95 (14.77-27.05)
TT ≥ 50 ng/dL in males (n, %)					
Yes	318 (47.4)	–	–	24 (7.5)	329 (93.7)
No	353 (52.6)	–	–	296 (92.5)	22 (6.3)
E2 ≥ 20 pg/ml in females (n, %)					
Yes	308 (43.3)	71 (22.0)	332 (85.3)	–	–
No	403 (56.7)	251 (78.0)	57 (14.7)	–	–
Puberty status (n, %)^6^					
Pubertal	806 (58.3)	71 (22.05)	382 (98.20)	24 (7.50)	329 (93.73)
Prepubertal	576 (41.7)	251 (77.95)	7 (1.80)	296 (92.50)	22 (6.27)
Dietary inflammatory index [mean (SD)]	0.92 (0.33)	0.89 (0.36)	0.95 (0.28)	0.93 (0.31)	0.86 (0.27)

^1^Statistics were unweighted; children (6–11 years) and adolescents (12–19 years).

^2^BMI category was defined as underweight (BMI < 5th percentile), normal weight (5th percentile ≤ BMI < 85th percentile), overweight (85th percentile ≤ BMI < 95th percentile), obese (BMI ≥ 95th percentile).

^3^Only 8 girls (6-11 years) and 132 female adolescents recorded information on “Age at first menstrual period”.

^4^Females who answered “Yes” to the question “menstrual periods started yet” or with available information on the age at first menstrual period were recognized as “menstrual periods started.”

^5^TT, total testosterone; E2, total estradiol; SHBG, sex hormone binding globulin; FAI, free androgen index, was calculated as total testosterone (ng/dL)/SHBG (nmol/L), TT/E2 was calculated as TT (ng/dL)/E2 (pg/ml).

^6^Puberty status was defined as “pubertal” if TT ≥ 50 ng/dL in males, E2 ≥ 20 pg/ml or menstrual period started in females, otherwise puberty status was defined as “prepubertal”.

**Table 2:** Distribution of sex hormones in different DII quantiles^1^.

Characteristics^2^	Mean(range)				P-value^a^
	Quartile 1 (n = 345)	Quartile 2 (n = 345)	Quartile 3 (n = 348)	Quartile 4 (n = 344)	
TT	16.20 (4.44-43.95)	20.00 (4.52-43.60)	16.50 (3.87-53.70)	23.45 (6.09-62.10)	0.005
FAI	0.32 (0.06-2.62)	0.32 (0.05-1.10)	0.25 (0.04-1.45)	0.50 (0.08-2.95)	0.031
E2	11.80 (2.11-30.90)	9.20 (2.11-29.90)	16.80 (2.11-38.75)	15.05 (2.11-38.75)	0.505
SHBG	54.61 (33.42-98.90)	60.34 (36.96-94.64)	59.20 (34.52-100.30)	53.05 (32.83-89.41)	0.011
TT/E2	1.10 (0.52-5.37)	1.31 (0.64-7.65)	1.58 (0.61-8.24)	1.69 (0.64-8.90)	0.010

^1^Quartile 1: -5.42 to -0.03; Quartile 2: -0.02 to 1.18; Quartile 3: 1.19 to 2.18; Quartile 4: 2.19 to 4.39.

^2^TT, total testosterone; E2, total estradiol; SHBG, sex hormone binding globulin; FAI, free androgen index, was calculated as total testosterone (ng/dL)/SHBG (nmol/L), TT/E2 was calculated as TT (ng/dL)/E2 (pg/ml).

a Kruskal-Wallis test was used to compare the differences in sex hormone levels between different DII groups.

**Table 3** shows the association between consecutive DII and sex hormone indicators by gender and age group. In male adolescents, DII is always negatively associated with TT (P-trend = 0.09), FAI (P-trend = 0.03) and E2 (P-trend = 0.01), and monotonically positively associated with SHBG (P-trend = 0.02). In female adolescents, with the increase of DII, significant positive associations with SHBG were observed (β 0.017, 95%CI: 0.009,0.053) (**Table 3**). In addition, DII was negatively associated with TT (P-trend = 0.01) and FAI (P-trend = 0.02) and showed a monotonous downward trend in the quartile of exposure.

**Table 3:** Association between categorical dietary inflammatory index (DII) and sex hormones by sex-age groups in 6-19-year old participants in NHANES 2015-2016.

Sex	Subgroup	DII	TT β (95%CI)	FAI β (95%CI)	E2 β (95%CI)	SHBG β (95%CI)	TT/E2 β (95%CI)
Female	Children	Q1	Reference	Reference	NA	Reference	NA
		Q2	0.063 (-0.126,0.082)	-0.013 (-0.069,0.103)	NA	-0.019 (-0.006,0.125)	NA
		Q3	-0.092 (-0.162,0.012)	0.031 (-0.136,0.163)	NA	0.029 (-0.012,0.103)	NA
		Q4	-0.186 (-0.102,0.129)	-0.098 (-0.169,0.052)	NA	-0.272 (-0.598,0.029)	NA
	Adolescents	Q1	Reference	Reference	Reference	Reference	Reference
		Q2	-0.009 (-0.109,0.016)	-0.026 (-0.083,0.162)	-0.076 (-0.106,0.096)	-0.016 (-0.103,0.083)	-0.012 (-0.083,0.036)
		Q3	-0.012 (-0.038,0.017)	-0.010 (-0.083,0.032)	-0.039 (-0.065,0.326)	0.031 (0.012,0.282)*	-0.041 (-0.076,0.012)
		Q4	-0.031 (-0.053,-0.006)*	-0.032 (-0.063,-0.003)	-0.062 (-0.083,-0.003)*	0.052 (0.021,0.096)*	-0.056 (-0.381,0.016)
Male	Children	Q1	Reference	Reference	NA	Reference	NA
		Q2	-0.013 (-0.352,0.012)	0.013 (-0.082,0.063)	NA	-0.035 (-0.082,0.106)	NA
		Q3	-0.029 (-0.042,0.009)	-0.063 (-0.131,0.023)	NA	-0.083 (-0.103,0.063)	NA
		Q4	-0.057 (-0.063,0.015)	-0.032 (-0.092,0.103)	NA	0.013 (-0.016,0.062)	NA
	Adolescents	Q1	Reference	Reference	Reference	Reference	Reference
		Q2	-0.016 (-0.032,0.009)	0.012 (-0.016,0.009)	-0.029 (-0.072,0.013)	-0.063 (-0.106,0.082)	-0.023 (-0.069,0.025)
		Q3	-0.023 (-0.037,0.013)	-0.035 (-0.062,0.012)	-0.042 (-0.068,0.026)	0.013 (0.003,0.037)*	-0.067 (-0.126,0.012)
		Q4	-0.083 (-0.035,-0.016)*	-0.083 (-0.038,0.026)*	-0.086 (-0.136,-0.025)*	0.017 (0.009,0.053)*	-0.093 (-0.135,0.026)

Children (6-11 years) and adolescents (12-19 years). Covariate-adjusted multiple linear regression was used to assess the association between categorical dietary inflammatory index(DII) and sex hormones by sex-age groups in 6-19-year old participants. Estimates were presented as coefficients and 95% confidence intervals (CIs) and were adjusted for urinary creatinine, age, race/ethnicity, BMI category, ratio of family income to poverty, time of sample collection. “NA” indicates the estimates were not available in children as the detection frequency of E2 was < 50% in that group.

*P < 0.05.

In children, as the quartile of DII increases, there is no consistent trend for sex hormones, and there are few significant associations. Only in male children, DII (4th and 1st quantile) was significantly negatively associated with TT, and significantly positively associated with SHBG. At the same time, among female children, DII was significantly negatively associated with FAI (fourth quartile vs the first quartile) and positively associated with SHBG (fourth quartile vs the first quartile) (**Table 3**).

The associations and patterns of the DII quartiles of puberty status in men and women with sex hormones are similar to the associations and patterns of gender and age groups, but some differences have been found. Specifically, in pubertal males, DII was consistent and significantly negatively associated with FAI and E2, and positively associated with SHBG and TT/E2. Among pubertal females, significant negative associations between DII and TT and a significant positive association between SHBG were observed in this group (**Table 4**). Similar to the results for children, there is very little significant association between DII and sex hormones in prepubertal individuals. DII is positively associated with SHBG of prepubertal males and negatively associated with FAI of female prepubertal.

**Table 4:** Association between categorical dietary inflammatory index (DII) and sex hormones by sex-puberty status groups in 6-19-year old participants in NHANES 2015-2016.

Sex	Subgroup	DII	TT β (95%CI)	FAI β (95%CI)	E2 β (95%CI)	SHBG β (95%CI)	TT/E2 β (95%CI)
Female	Prepubertal	Q1	Reference	Reference	NA	Reference	NA
		Q2	0.026 (-0.203,0.102)	0.035 (-0.023,0.063)	NA	-0.031 (-0.062,0.103)	NA
		Q3	-0.016 (-0.063,0.032)	-0.023 (-0.062,0.031)	NA	-0.065 (-0.093,0.042)	NA
		Q4	-0.063 (-0.083,0.026)	-0.043 (-0.081,-0.012)^*^	NA	-0.083 (-0.102,0.015)	NA
	Pubertal	Q1	Reference	Reference	Reference	Reference	Reference
		Q2	-0.015 (-0.109,0.016)	-0.019 (-0.062,0.072)	-0.053 (-0.136,0.092)	-0.009 (-0.026,0.013)	-0.016 (-0.083,0.006)
		Q3	-0.102 (-0.238,0.137)	-0.023 (-0.102,0.093)	-0.082 (-0.239,0.041)	0.022 (0.013,0.046)^*^	-0.027 (-0.053,0.013)
		Q4	-0.089 (-0.132,-0.031)^*^	-0.052 (-0.162,0.013)	-0.203 (-1.327,0.092)	0.016 (0.009,-0.032)^*^	-0.063 (-0.092,0.026)
Male	Prepubertal	Q1	Reference	Reference	NA	Reference	NA
		Q2	-0.019 (-0.042,0.011)	-0.013 (-0.082,0.016)	NA	-0.036 (-0.072,0.025)	NA
		Q3	-0.035 (-0.053,0.008)	-0.019 (-0.031,0.023)	NA	-0.023 (-0.051,0.013)	NA
		Q4	-0.039 (-0.062,0.016)	-0.028 (-0.039,0.019)	NA	0.009 (0.003,0.026)^*^	NA
	Pubertal	Q1	Reference	Reference	Reference	Reference	Reference
		Q2	-0.013 (-0.035,0.012)	-0.012 (-0.026,0.002)	-0.019 (-0.026,0.006)	-0.027 (-0.048,0.013)	-0.019 (-0.037,0.008)
		Q3	-0.019 (-0.042,0.021)	-0.022 (-0.053,-0.009)^*^	-0.023 (-0.051,0.013)	0.012 (-0.009,0.027)^*^	-0.023 (-0.036,0.016)
		Q4	-0.032 (-0.051,0.002)	-0.039 (-0.063,-0.015)^*^	-0.037 (-0.062,-0.012)^*^	0.035 (0.016,0.056)^*^	-0.036 (-0.052,-0.012)^*^

Puberty status was defined as “pubertal” if TT ≥ 50 ng/dL in males, E2 ≥ 20 pg/ml or menstrual period started in females, otherwise puberty status was defined as “prepubertal”. Covariate-adjusted multiple linear regression was used to assess the association between categorical dietary inflammatory index(DII) and sex hormones by sex-puberty status groups in 6-19-year old participants. Estimates were presented as coefficients and 95% confidence intervals (CIs) and were adjusted for urinary creatinine, age, race/ethnicity, BMI category, ratio of family income to poverty, time of sample collection. “NA” indicates the estimates were not available in prepubertal participants as the detection frequency of E2 was < 50% in that group.

*P < 0.05.

## Discussion

Our study is the first to investigate the association between DII and sex steroids in participants aged 6-19 years. We found that DII was significantly negatively associated with sex steroids in children or adolescent individuals, and positively associated with SHBG and TT/E2. However, these associations are gender-dependent to some extent, especially among adolescents and adolescents. The associations between TT and/or FAI observed in men are consistent and stronger. In contrast, there is little evidence that there is a significant association between DII and sex steroids in children or prepubertal individuals (29).

To the best of our knowledge, this is the first study to evaluate the association between the inflammatory potential of the overall diet pattern and sex hormones. Emerging evidence suggests that one possible mechanism for our results may be the influence of diet on pro-inflammatory markers such as IL-1, IL-6, IL-17, and tumor necrosis factor. These markers severely impair the secretion of sex hormones by activating inflammation and the production of reactive oxygen species in interstitial macrophages adjacent to Leydig cells (30, 31). In addition, some experimental studies have also shown that these pro-inflammatory cytokines, including IL-6, IL-1b, and tumor necrosis factor-a, can regulate the hypothalamic-pituitary-gonadal axis to inhibit the secretion of sex hormones (32, 33). For children and adolescents, excessive visceral adipose tissue is a cause of chronic inflammation, because adipose tissue is the main source of pro-inflammatory mediators. Under inflammatory conditions, more adipose tissue produces aromatase, which converts T into E2, thereby reducing T levels. According to reports, excessive inflammation may cause negative effects on Leydig cell function and reduce the production of sex hormones (particularly sensitive to inflammation), so it is reasonable to think that anti-inflammatory treatments can prevent sex hormone abnormalities (34, 35). Our findings indicate that an anti-inflammatory diet may be a viable way to reduce the burden of accumulated inflammation.

Studies have reported that in animal experiments, inflammatory cytokines significantly increased the plasma E2 concentration of female animals, but did not change the level of testosterone, while in male animals, the level of testosterone decreased and the level of E2 increased. Further exploration of research, the results revealed that in male animals, inflammatory cytokines lead to a significant up-regulation of gonadotropin-releasing hormone and down-regulation of luteinizing hormone in the brain and receptors in the testes (36, 37). At the same time, key genes that regulate the pathway of steroid production have also been observed in male animals, which may include 17β-hydroxysteroid dehydrogenase (17β-HSD), steroid production acute regulatory protein (star), and hydroxymethylglutaryl-CoA Significant down-regulation of reductase (hmgra). These changes are related to the up-regulation of cytochrome P450 corresponding enzymes. In contrast, in female fish, down-regulation of gonadotropin-releasing hormone receptors in the brain and up-regulation of follicle-stimulating hormone and luteinizing hormone receptors were observed in female fish and the transcription up-regulation of luteinizing hormone receptors and most steroid-producing genes in the ovaries. Because these enzymes and receptors play a key role in steroid production, their down-regulation and the up-regulation of aromatase encoding genes in male animals may partly explain the significant reduction in male androgen levels (TT or FAI) in adolescents and adolescents compared to females in our study (38, 39).

In fact, in our study, the negative associations between DII and E2 is related to the reduction of TT and/or FAI levels. Since E2 is mainly converted from TT and other androgens through aromatase, we speculate that the decrease in E2 may be the result of the decrease in precursors. At the same time, the increase in DII may inhibit aromatase activity, because we have observed some suggestive positive associations between DII and TT/E2 in adolescent boys and female adolescents, which may further lead to negative associations with E2 (40, 41). However, the assessment of aromatase activity based on the TT/E2 ratio may be inaccurate, and the significant association with TT/E2 may partly be due to changes in E2, making this assumption uncertain. In addition, we are not aware of any studies that provide direct evidence to explain the negative associations with E2 observed in adolescent and adolescent individuals. Further research is needed to confirm our findings and explore possible mechanisms (41–44).

Additionally, we discovered that DII is positively associated with SHBG in adolescents and adolescents. As we all know, SHBG possesses a high affinity for binding to testosterone and E2. It is the main plasma protein from the liver and provides a circulating steroid hormone pool. Therefore, the general standard of SHBG mainly affects the circulating levels of testosterone and E2 in free form, However, SHBG production is quoque partially inhibited by testosterone due to E2 stimulation. All these internal relationships support that the negative associations between TT and FAI is accompanied by the positive associations with SHBG. In contrast to adolescents and adolescent individuals, the association between DII and sex hormones in children and prepubertal individuals is generally not significant. This may be on account of pre-adolescent child sex steroid hormone levels are very low (45, 46).

Our research has many advantages. It is worth noting that we conducted a subgroup analysis of different age stages and adolescent states and found that the association between DII and sex hormone levels is significant in puberty and adolescents. In addition, isotope dilution liquid chromatography and tandem mass spectrometry are used to measure TT and E2 levels, providing more sensitive results than immunoassays. However, the main limitation of the current design is that NHANES is a cross-sectional database, which severely hinders our ability to make causal inferences. Secondly, dietary intake is self-reported, with recall bias, estimated based on 24-hour history, and cannot reflect daily changes in dietary intake. Another potential limitation is that out of the possible 45 food parameters, only 27 food parameters can be used for DII calculations, and previous studies have successfully verified the modified DII (27 items) in NHANES. Nonetheless, these unbalanced 27 items (because they are not divided equally into pro-inflammatory and anti-inflammatory) may also erroneously distort the inflammatory potential of the diet (7, 47). Finally, serum sex hormones are only measured at a single time point, while the AUA guidelines recommend 2 levels because of the intra-individual and diurnal variation of serum sex hormones.

## Conclusions

In summary, DII was significantly and inversely associated with TT and (or) FAI and E2 and positively associated with SHBG in both female and male adolescents or pubertal individuals. The associations were also sometimes sex-dependent, with consistent and stronger associations of TT and FAI being observed in male adolescents and pubertal boys. In contrast, the associations in children or prepubertal individuals were almost non-significant. Given the cross-sectional nature of the research design and the small sample size, more research is needed to confirm our findings.

## Data Availability Statement

Publicly available data sets were analyzed in this study. These data can be found here: NHANES https://www.cdc.gov/nchs/nhanes/index.htm.

## Ethics Statement

The studies involving human participants were reviewed and approved by the NCHS Ethic Review Board. Written informed consent to participate in this study was provided by the participants’ legal guardian/next of kin.

## Author Contributions

YM, RL: project development, data collection, management and analysis, manuscript writing. WZ, YZ, YS: Project development, data management and analysis, manuscript writing and editing. HT, HZ, XH: Data collection, management, and analysis. RL, BY: Data analysis, manuscript editing. All authors contributed to the article and approved the submitted version.

## Funding

The Community Cohort Study on Specialized Nervous System Diseases (2017YFC0907701).

## Conflict of Interest

The authors declare that the research was conducted in the absence of any commercial or financial relationships that could be construed as a potential conflict of interest.

## Publisher’s Note

All claims expressed in this article are solely those of the authors and do not necessarily represent those of their affiliated organizations, or those of the publisher, the editors and the reviewers. Any product that may be evaluated in this article, or claim that may be made by its manufacturer, is not guaranteed or endorsed by the publisher.

## References

[1] Kaluza J Harris H Melhus H Michaëlsson K Wolk A. Questionnaire-Based Anti-Inflammatory Diet Index as a Predictor of Low-Grade Systemic Inflammation. Antioxid Redox Signal (2018) 28:78–84. doi: 10.1089/ars.2017.7330 28877589

[2] Hart MJ Torres SJ McNaughton SA Milte CM. Dietary Patterns and Associations With Biomarkers of Inflammation in Adults: A Systematic Review of Observational Studies. Nutr J (2021) 20:24. doi: 10.1186/s12937-021-00674-9 33712009 PMC7955619

[3] Ozawa M Shipley M Kivimaki M Singh-Manoux A Brunner EJ. Dietary Pattern, Inflammation and Cognitive Decline: The Whitehall II Prospective Cohort Study. Clin Nutr (2017) 36:506–12. doi: 10.1016/j.clnu.2016.01.013 PMC5381339 26874911

[4] Mendes FC Paciência I Cavaleiro RJ Farraia M Silva D Padrão P. Higher Diversity of Vegetable Consumption Is Associated With Less Airway Inflammation and Prevalence of Asthma in School-Aged Children. Pediatr Allergy Immunol (2021) 32:925–36. doi: 10.1111/pai.13446 33394508

[5] Hosseini B Berthon BS Saedisomeolia A Starkey MR Collison A Wark PAB. Effects of Fruit and Vegetable Consumption on Inflammatory Biomarkers and Immune Cell Populations: A Systematic Literature Review and Meta-Analysis. Am J Clin Nutr (2018) 108:136–55. doi: 10.1093/ajcn/nqy082 29931038

[6] Kotemori A Sawada N Iwasaki M Yamaji T Shivappa N Hebert JR. Dietary Inflammatory Index Is Associated With Inflammation in Japanese Men. Front Nutr (2021) 8:604296. doi: 10.3389/fnut.2021.604296 33898494 PMC8062774

[7] Shivappa N Steck SE Hurley TG Hussey JR Hebert JR. Designing and Developing a Literature-Derived, Population-Based Dietary Inflammatory Index. Publ Health Nutr (2014) 17:1689e96. doi: 10.1017/S1368980013002115 PMC3925198 23941862

[8] Shivappa N Hebert JR Rietzschel ER De Buyzere ML Langlois M Debruyne E. Associations Between Dietary Inflammatory Index and Inflammatory Markers in the Asklepios Study. Br J Nutr (2015) 113:665e71. doi: 10.1017/S000711451400395X 25639781 PMC4355619

[9] Wood LG Shivappa N Berthon BS Gibson PG Hebert JR. Dietary Inflammatory Index Is Related to Asthma Risk, Lung Function and Systemic Inflammation in Asthma. Clin Exp Allergy (2015) 45:177–83. doi: 10.1111/cea.12323 PMC4190104 24708388

[10] Hirko KA Spiegelman D Barnett JB Cho E Willett WC Hankinson SE. Dietary Patterns and Plasma Sex Hormones, Prolactin, and Sex Hormone-Binding Globulin in Premenopausal Women. Cancer Epidemiol Biomarkers Prev (2016) 25:791–8. doi: 10.1158/1055-9965.EPI-15-1019 PMC5082703 26980437

[11] AlEssa HB Malik VS Yuan C Willett WC Huang T Hu FB. Dietary Patterns and Cardiometabolic and Endocrine Plasma Biomarkers in US Women. Am J Clin Nutr (2017) 105:432–41. doi: 10.3945/ajcn.116.143016 PMC5267305 27974312

[12] Hirko Kelly A Spiegelman D Barnett JB Cho E Willett WC Hankinson SE. Dietary Patterns and Plasma Sex Hormones, Prolactin, and Sex Hormone-Binding Globulin in Premenopausal Women. Cancer Epidemiol Biomarkers Prev (2016) 25:791–8. doi: 10.1158/1055-9965.EPI-15-1019 PMC5082703 26980437

[13] Qin Z Liu N Liao R Jiang L Su B. The Association Between Dietary Inflammatory Potential and Sex Hormones in Male Children and Adolescents Aged 6-19 Years. Front Endocrinol (Lausanne) (2021) 12:722941. doi: 10.3389/fendo.2021.722941 34413832 PMC8370775

[14] Varlamov O. Western-Style Diet, Sex Steroids and Metabolism. Biochim Biophys Acta (BBA)-Molecular Basis Dis (2017) 1863(5):1147–55. doi: 10.1016/j.bbadis.2016.05.025 27264336

[15] Mayr HL Itsiopoulos C Tierney AC Ruiz-Canela M Hebert JR Shivappa N. Improvement in Dietary Inflammatory Index Score After 6-Month Dietary Intervention Is Associated With Reduction in Interleukin-6 in Patients With Coronary Heart Disease: The AUSMED Heart Trial. Nutr Res (2018) 55:108–21. doi: 10.1016/j.nutres.2018.04.007 29807669

[16] Obón-Santacana M Romaguera D Gracia-Lavedan E Molinuevo A Molina-Montes E Shivappa N. Dietary Inflammatory Index, Dietary Non-Enzymatic Antioxidant Capacity, and Colorectal and Breast Cancer Risk (MCC-Spain Study). Nutrients (2019) 11(6):1406. doi: 10.3390/nu11061406 PMC6628286 31234427

[17] Shin D Lee KW Brann L Shivappa N Hébert JR. Dietary Inflammatory Index Is Positively Associated With Serum High-Sensitivity C-Reactive Protein in a Korean Adult Population. Nutrition (2019) 63:155–61. doi: 10.1016/j.nut.2018.11.016 30999247

[18] Shin PK Park SJ Kim MS Kwon DY Kim MJ Kim K. A Traditional Korean Diet With a Low Dietary Inflammatory Index Increases Anti-Inflammatory IL-10 and Decreases Pro-Inflammatory NF-κb in a Small Dietary Intervention Study. Nutrients (2020) 12(8):2468. doi: 10.3390/nu12082468 PMC7468714 32824387

[19] Bujtor M Turner AI Torres SJ Esteban-Gonzalo L Pariante CM Borsini A. Associations of Dietary Intake on Biological Markers of Inflammation in Children and Adolescents: A Systematic Review. Nutrients (2021) 13(2):356. doi: 10.3390/nu13020356 33503979 PMC7911843

[20] Lumley T Scott A. Fitting Regression Models to Survey Data. Stat Sci (2017) 32(2):265–78. doi: 10.1214/16-STS605

[21] Lewis RC Johns LE Meeker JD. Serum Biomarkers of Exposure to Perfluoroalkyl Substances in Relation to Serum Testosterone and Measures of Thyroid Function Among Adults and Adolescents From NHANES 2011–2012. Int. J. Environ. Res. Public Health (2015) 12:6098–114. doi: 10.3390/ijerph120606098 PMC4483690 26035660

[22] Lewis RC Meeker JD. Biomarkers of Exposure to Molybdenum and Other Metals in Relation to Testosterone Among Men From the United States National Health and Nutrition Examination Survey 2011–2012. Fertil Steril (2015) 103:172–8. doi: 10.1016/j.fertnstert.2014.09.020 PMC4282605 25439796

[23] Shivappa N Steck SE Hurley TG Hussey JR Ma Y Ockene IS. A Population-Based Dietary Inflammatory Index Predicts Levels of C-Reactive Protein in the Seasonal Variation of Blood Cholesterol Study (SEASONS). Public Health Nutr (2014) 17:1825–33. doi: 10.1017/S1368980013002565 PMC3983179 24107546

[24] Tabung FK Smith-Warner SA Chavarro JE Wu K Fuchs CS Hu FB. Development and Validation of an Empirical Dietary Inflammatory Index. J Nutr (2016) 146:1560–70. doi: 10.3945/jn.115.228718 PMC4958288 27358416

[25] Mazidi M Shivappa N Wirth MD Hebert JR Vatanparast H Kengne AP. The Association Between Dietary Inflammatory Properties and Bone Mineral Density and Risk of Fracture in US Adults. Eur J Clin Nutr (2017) 71:1273–7. doi: 10.1038/ejcn.2017.133 29019343

[26] NCHS. Laboratory Procedure Manual for Serum Sex Hormone Binding Globulin. National Center for Environmental Health (2013-2014a). Available at: https://wwwn.cdc.gov/nchs/data/nhanes/2013-2014/labmethods/TST_H_MET_Sex_Hormone_Binding_Globulin.pdf.

[27] NCHS. Laboratory Procedure Manual for Serum Total Estradiol and Total Testosterone. National Center for Environmental Health (2013-2014b). Available at: https://wwwn.cdc.gov/nchs/data/nhanes/2013-2014/labmethods/TST_H_MET_Total_Estradiol_and_Total_Testosterone.pdf.

[28] Haghighatdoost F Feizi A Esmaillzadeh A Feinle-Bisset C Keshteli AH Afshar H. Association Between the Dietary Inflammatory Index and Common Mental Health Disorders Profile Scores. Clin Nutr (2019) 38:1643–50. doi: 10.1016/j.clnu.2018.08.016 30190117

[29] Qin Z Liu N Liao R Jiang L Su B. The Association Between Dietary Inflammatory Potential and Sex Hormones in Male Children and Adolescents Aged 6-19 Years. Front Endocrinol (Lausanne) (2021) 12:722941. doi: 10.3389/fendo.2021.722941 34413832 PMC8370775

[30] Tremellen K McPhee N Pearce K Benson S Schedlowski M Engler H. Endotoxin-Initiated Inflammation Reduces Testosterone Production in Men of Reproductive Age. Am J Physiol Endocrinol Metab (2018) 314:E206–13. doi: 10.1152/ajpendo.00279.2017 PMC5899218 29183872

[31] Uchoa Mariana F Moser VA Pike CJ. Interactions Between Inflammation, Sex Steroids, and Alzheimer’s Disease Risk Factors. Front Neuroendocrinol (2016) 43:60–82. doi: 10.1016/j.yfrne.2016.09.001 27651175 PMC5123957

[32] Calder PC Ahluwalia N Brouns F Buetler T Clement K Cunningham K. Dietary Factors and Low-Grade Inflammation in Relation to Overweight and Obesity. Br J Nutr (2011) 106(S3):S1–S78. doi: 10.1017/S0007114511005460 22133051

[33] Fui MNgT Dupuis P Grossmann M. Lowered Testosterone in Male Obesity: Mechanisms, Morbidity and Management. Asian J Androl (2014) 16:223–31. doi: 10.4103/1008-682X.122365 PMC3955331 24407187

[34] Tremellen K McPhee N Pearce K. Metabolic Endotoxaemia Related Inflammation Is Associated With Hypogonadism in Overweight Men. Basic Clin Androl (2017) 27:5. doi: 10.1186/s12610-017-0049-8 28286655 PMC5341351

[35] Bini EI D’Attilio L Marquina-Castillo B Mata-Espinosa D Díaz A Marquez-Velasco R. The Implication of Pro-Inflammatory Cytokines in the Impaired Production of Gonadal Androgens by Patients With Pulmonary Tuberculosis. Tuberculosis (Edinb) (2015) 95:701–6. doi: 10.1016/j.tube.2015.06.002 26602224

[36] Pradhan A Olsson P-E. Zebrafish Sexual Behavior: Role of Sex Steroid Hormones and Prostaglandins. Behav Brain Funct (2015) 11:23. doi: 10.1186/s12993-015-0068-6 26385780 PMC4575480

[37] Wang YC Li N Zhao Y Zhang LJ. Effects of Female Sex Hormones on Chemotherapeutic Paclitaxel-Induced Neuropathic Pain and Involvement of Inflammatory Signal. J Biol Regul Homeost Agents (2018) 32:1157–63. 30334407

[38] Melo AF Jr Dalpiaz PLM Escouto LDS Sousa GJ Aires R Oliveira ND. Involvement of Sex Hormones, Oxidative Stress, ACE and ACE2 Activity in the Impairment of Renal Function and Remodelling in SHR. Life Sci (2020) 257:118138. doi: 10.1016/j.lfs.2020.118138 32712298

[39] Alyoussef A Al-Gayyar MMH. Thymoquinone Ameliorated Elevated Inflammatory Cytokines in Testicular Tissue and Sex Hormones Imbalance Induced by Oral Chronic Toxicity With Sodium Nitrite. Cytokine (2016) 83:64–74. doi: 10.1016/j.cyto.2016.03.018 27038016

[40] Bi Y Jiang M Guo W Guan X Xu M Ren S. Sex-Dimorphic and Sex Hormone-Dependent Role of Steroid Sulfatase in Adipose Inflammation and Energy Homeostasis. Endocrinology (2018) 159:3365–77. doi: 10.1210/en.2018-00531 PMC6112598 30060148

[41] Leitner L Jürets A Itariu BK Keck M Prager G Langer F. Osteopontin Promotes Aromatase Expression and Estradiol Production in Human Adipocytes. Breast Cancer Res Treat (2015) 154:63–9. doi: 10.1007/s10549-015-3603-0 26482249

[42] Pedersen AL Brownrout JL Saldanha CJ. Central Administration of Indomethacin Mitigates the Injury-Induced Upregulation of Aromatase Expression and Estradiol Content in the Zebra Finch Brain. Endocrinology (2017) 158:2585–92. doi: 10.1210/en.2017-00346 PMC5551551 28575175

[43] Ge W Duan H Xiao L Lv J Jiang Y Ding Z. 17β-Estradiol Protects Sheep Oviduct Epithelial Cells Against Lipopolysaccharide-Induced Inflammation. Vitro Mol Immunol (2020) 127:21–30. doi: 10.1016/j.molimm.2020.08.016 32905905

[44] Wright CL Hoffman JH McCarthy MM. Evidence That Inflammation Promotes Estradiol Synthesis in Human Cerebellum During Early Childhood. Transl Psychiatry (2019) 9:58. doi: 10.1038/s41398-018-0363-8 30705253 PMC6355799

[45] Park B Lee Y-J. Inverse Association of Testosterone and Sex Hormone Binding Globulin Levels With Leukocyte Count in Middle-Aged and Elderly Men. Aging Male (2018) 21:176–81. doi: 10.1080/13685538.2018.1477934 29863448

[46] Yamazaki H Kushiyama A Sakoda H Fujishiro M Yamamotoya T Nakatsu Y. *In Vitro*Protective Effect of Sex Hormone-Binding Globulin Against Metabolic Syndrome : Evidence Showing Anti-Inflammatory and Lipolytic Effects on Adipocytes and Macrophages. Mediators Inflamm (2018) 2018:3062319. doi: 10.1155/2018/3062319 30046278 PMC6036814

[47] Shivappa N Wirth MD Hurley TG Hébert JR. Association Between the Dietary Inflammatory Index (DII) and Telomere Length and C-Reactive Protein From the National Health and Nutrition Examination Survey-1999–2002. Mol Nutr Food Res (2017) 61(4):1600630. doi: 10.1002/mnfr.201600630 PMC5380547 27878970

