# Supplementary figures and images for: Associations Between Dietary Inflammatory Index and Sex Hormones Among 6- to 19-Year-Old Children and Adolescents in NHANES 2015–2016
Source: Front Endocrinol (Lausanne). 2022 Jan 10;12:792114. doi: 10.3389/fendo.2021.792114 (PMC8784841; doi:10.3389/fendo.2021.792114)

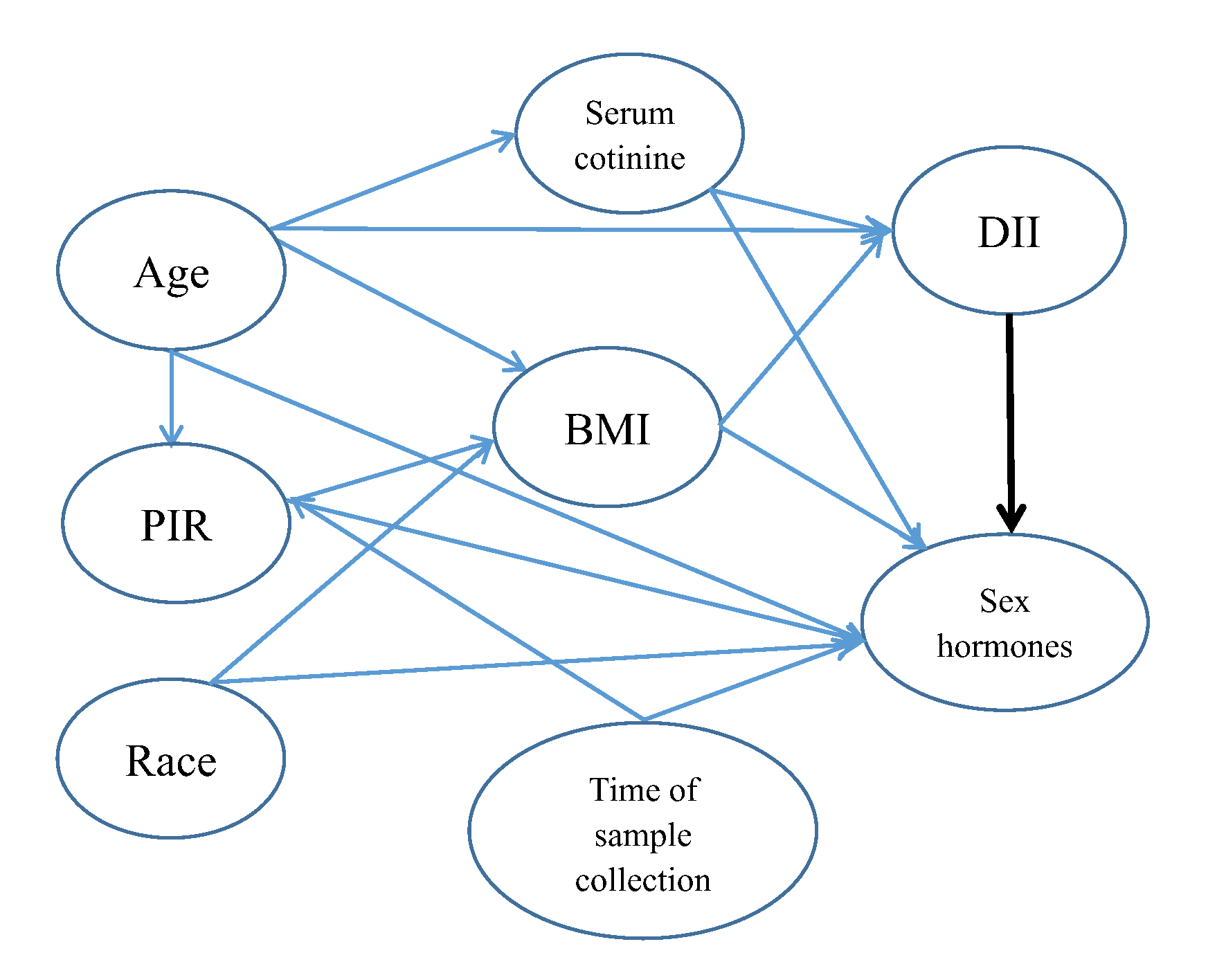

Supplement: Supplementary file 1 [file Image_1.tiff]
