# Supplementary material for: Associations Between Dietary Inflammatory Index and Sex Hormones Among 6- to 19-Year-Old Children and Adolescents in NHANES 2015–2016
Source: Front Endocrinol (Lausanne). 2022 Jan 10;12:792114. doi: 10.3389/fendo.2021.792114 (PMC8784841; doi:10.3389/fendo.2021.792114)
Supplement: Supplementary file 2 [file Table_1.docx]

Supplementary Table 1 Nutrient content of study participants.

| Characteristic | Mean±SD |
| --- | --- |
|  |  |
| **Carbohydrates (g/day)** | 233.21±94.33 |
| **Protein (g/day)** | 65.03±29.02 |
| **Total fat** **(g/day)** | 69.85±32.57 |
| **β-Carotene (μg/day)** | 923.74±1482.75 |
| **Fiber (g/day)** | 13.61±7.02 |
| **Cholesterol (mg/day)** | 213.98±156.06 |
| **Saturated fat (g/day)** | 24.17±12.92 |
| **Monounsaturated fat (g/day)** | 23.60±11.62 |
| **Polyunsaturated fats (g/day)** | 15.72±8.56 |
| **Niacin (mg/day)** | 20.64±9.83 |
| **Thiamine (mg/day)** | 1.48±0.71 |
| **Riboflavin (mg/day)** | 1.72±0.84 |
| **Vitamin B12 (μg/day)** | 4.12±2.72 |
| **Vitamin B6 (mg/day)** | 1.55±0.83 |
| **Fe (mg/day)** | 13.11±6.92 |
| **Magnesium (mg/day)** | 216.28±90.65 |
| **Zinc (mg/day)** | 9.29±4.81 |
| **Selenium (μg/day)** | 93.68±46.11 |
| **Vitamin A (RE/day)** | 493.79±333.69 |
| **Vitamin C (mg/day)** | 63.02±59.05 |
| **Vitamin E (mg/day)** | 6.63±3.89 |
| **Alcohol** **(g/day)** | 0.05±0.93 |
| **Omega 3** **fatty acid (g/day)** | 14.18±7.79 |
| **Omega 6 fatty acid (g/day)** | 1.39±0.85 |
| **Folic acid (μg/day)** | 188.72±146.09 |
